# Prolonged leisure time television watching as a risk factor for chronic obstructive pulmonary disease: Insights from Mendelian randomization

**DOI:** 10.1097/MD.0000000000042142

**Published:** 2025-04-18

**Authors:** Ying Li, Qingyi Zhou, Yuqing Cheng, Lianying Guo, Ye Yu, Mengqi Jiang, Lili Deng, Lu Sun, Xu Feng, Zhuo Zhang

**Affiliations:** a School of Public Health, Shenyang Medical College, Shenyang, China; b Department of Radiation Health Center, Liaoning Provincial Center for Disease Control and Prevention, Shenyang, China.

**Keywords:** causality, chronic obstructive pulmonary disease, Mendelian randomization, time spent watching television

## Abstract

Leisure sedentary behaviors are associated with an increased risk of chronic obstructive pulmonary disease (COPD), but whether this relationship is causal remains unknown. This study aimed to identify genetic determinants associated with leisure sedentary behaviors and estimate their potential causal effect on COPD risk. COPD case-control data were obtained from the Finnish biobank. Genome wide association analyses of leisure television watching, leisure computer use, and driving behavior in the UK Biobank identify 110, 82 and 6 genetic loci (*P* ＜ 5 × 10^−8^), respectively. A 2-sample Mendelian randomization (MR) analysis estimated a causal relationship between a 1.5-hour increase in television watching and a rise in COPD risk (OR = 2.725, 95% CI = 1.989–3.777, *P = *7.113 × 10^−10^). This relationship persisted independently of age at smoking initiation, daily cigarette consumption, educational years, and body mass index in comprehensive MR analyses. However, multivariate MR analyses showed that genetically predicted leisure time spent on computers and driving did not robustly influence COPD risk. In conclusion, this MR study suggests that a genetic predisposition for prolonged time spent watching television significantly increases the risk of COPD, corroborating findings from observational studies.

## 1. Introduction

Chronic obstructive pulmonary disease (COPD) is characterized by persistent airflow obstruction and ranks as the third leading cause of death globally.^[1,2]^ Recent epidemiological studies reveal that 20% to 30% of COPD cases occur in nonsmokers, suggesting the influence of additional risk factors.^[3,4]^ This observation emphasizes the necessity to explore other potential contributors, such as genetic predispositions and lifestyle changes in the development of COPD.^[5]^

Sedentary behavior, defined as any waking activity with an energy expenditure of ≤1.5 metabolic equivalents, typically involves sitting, reclining, or leaning.^[6]^ The prevalence of a sedentary lifestyle, marked notably by increased time spent watching television and using computers, represents a significant characteristic of modern society.^[7]^ Television watching, the predominant sedentary activity during adult leisure time,^[8]^ is extensively linked to detrimental health outcomes, including respiratory and cardiovascular diseases and certain cancers.^[9–12]^ Observational studies frequently use television watching as a proxy for overall leisure sedentary behavior due to its occurrence outside occupational settings, its susceptibility to intervention.^[13]^ This behavior is hypothesized to pose the most significant risk for COPD, associated with minimal and brief interruptions, reduced total energy expenditure, and distinct snacking habits that may exacerbate the adverse effects of prolonged sitting.^[14,15]^

Despite these associations, the causal relationship between sedentary behavior and COPD risk has not been thoroughly investigated. Our study employs Mendelian randomization (MR) to rigorously assess this connection, advancing the limitations of previous observational studies,^[16,17]^ which were subject to biases from confounding factors and reverse causation.^[18]^ By employing genetic variations as instrumental variables, the MR approach seeks to offer a more accurate assessment of the causal relationship.^[19]^

## 2. Methods

### 2.1. Study design and data source

**Figure 1.**
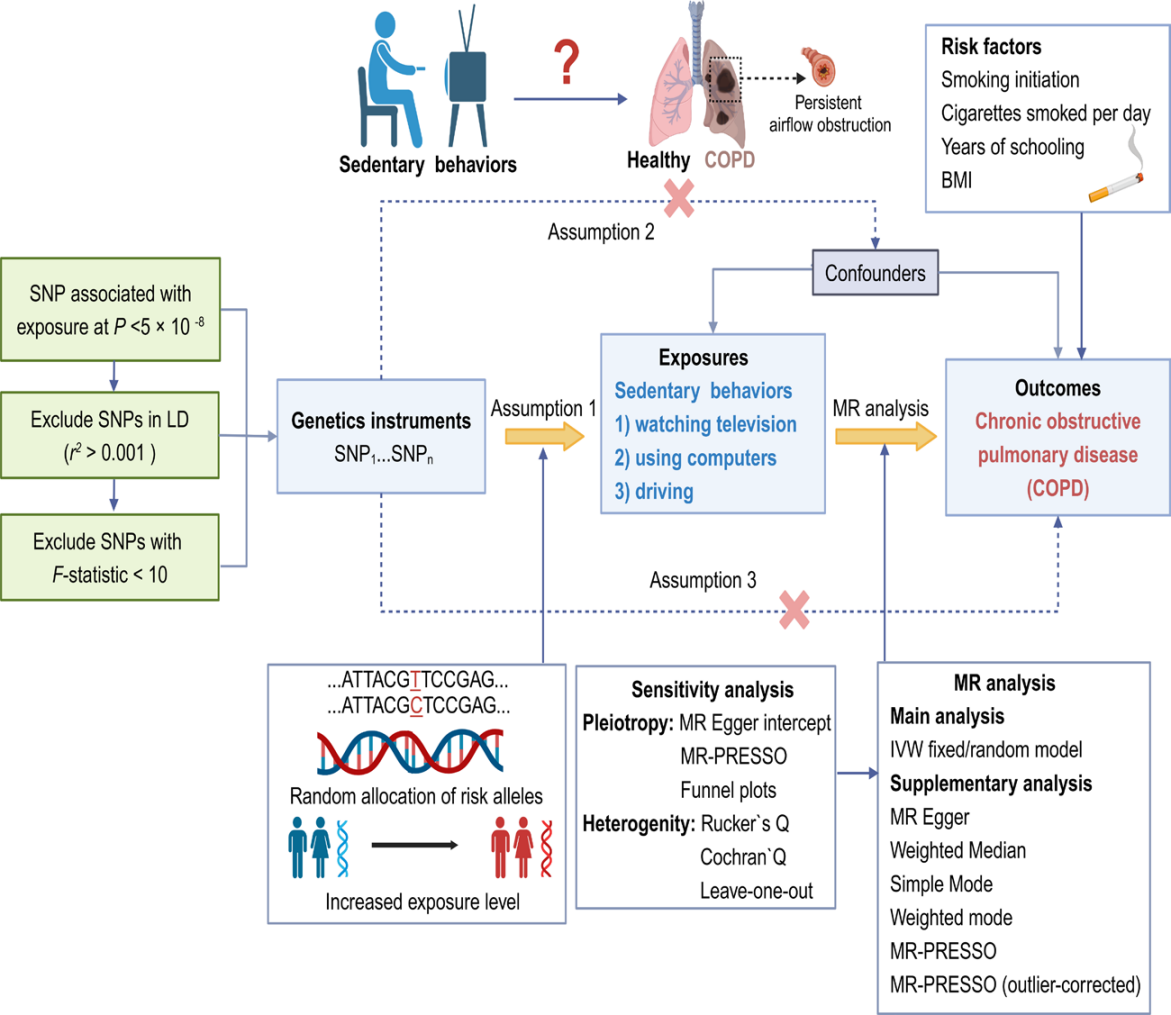
Overview of research design and MR design assumptions. Assumption 1 states that the genetic variation suggested as an instrumental variable must have a strong correlation with the risk factor being studied. Assumption 2 posits that this genetic variation should not have any correlation with any confounding influences. Assumption 3 posits that the selected genetic variation impacts the likelihood of the result solely through the risk factor, without exerting any influence on other routes. BMI = body mass index, LD = linkage disequilibrium, MR = Mendelian randomization. Created in BioRender (https://BioRender.com/oxgpeuq).

This study employs univariate and multivariate MR analyses to evaluate the potential causal impact of leisure sedentary behaviors, which include watching television, using computers, and driving, on the risk of COPD (Fig. 1). The cohort consisted of 54.3% women with an average age of 57.4 years.^[20]^ Time spent on each activity was assessed through responses to the following: “How many hours do you spend watching television on a typical day?” “How many hours do you spend using a computer on a typical day (excluding work-related use)?” and “How many hours do you spend driving on a typical day?” The study reported average daily times of 2.8 hours (standard deviation [SD] 1.5 hours) for television watching, 1.0 hours (SD 1.2 hours) for computer use, and 0.9 hours (SD 1.0 hours) for driving. Genetic variants linked to leisure sedentary behavior were adjusted for age, sex, body mass index (BMI), smoking status, hypertension, diabetes, Townsend deprivation index, physical activity levels, weekly alcohol use, and years of education. Additional information about the GWAS data is available in the previously published cohort profile.^[20]^

For COPD, GWAS summary data comprising 13,530 cases and 454,945 controls, totaling 24,180,654 single nucleotide polymorphisms (SNPs), were sourced from Finngen (https://r8.finngen.fi/). This study exclusively involved participants of European descent to reduce bias related to population stratification. Our multivariate analysis, acknowledging previous research, incorporated confounding factors closely associated with COPD risk: age at smoking initiation, cigarettes smoked per day,^[21–23]^ years of schooling,^[20,22]^ and BMI.^[24,25]^ The summary-level data on the age of smoking initiation and cigarettes smoked per day were extracted from the Sequencing Consortium of Alcohol and Nicotine Use Consortium (GSCAN), where phenotypes reflect smoking status and cigarettes smoked per day capture smoking intensity.^[26]^ Data on years of schooling came from a GWAS meta-analysis by the Social Science Genetic Association Consortium (SSGAC).^[27]^ BMI data was sourced from The UK Biobank by Genetic Investigation of Anthropometric Traits (GIANT), which included 681,275 participants. Given the public availability of this data, our study did not necessitate ethical approval (Table S1, Supplemental Digital Content, https://links.lww.com/MD/O710).

### 2.2. Selection of genetic instrument

The selection of effective genetic instruments was guided by 3 critical criteria: (1) a robust and consistent association with environmental exposures to leisure sedentary behaviors; (2) no direct correlation with COPD prevalence outcome; (3) independence from other confounding factors related to leisure sedentary behaviors and COPD. We selected genetic instruments using a relaxed threshold (*P* ＜ 5 × 10^−8^). We also analyzed the linkage disequilibrium (LD) of the chosen SNPs, identified independent SNPs (LD *r*^*2*^ ＜.001, window size 10,000 kb), and applied the PLINK clumping method to exclude dependent SNPs. To mitigate bias arising from weak instrumental variables, we calculated and then excluded instruments with an *F* -statistic ＜10 based on the formula: *F* = (β/SE)².^[28]^

### 2.3. Pleiotropy and sensitivity analysis

In our MR analysis, we utilized the inverse variance weighted (IVW) method, augmented by various sensitivity analyses (MR-Egger, weighted median, simple mode, weighted mode, and mendelian randomization pleiotropy residual sum and outlier [MR-PRESSO]) to increase the robustness of our findings.^[29]^ The IVW method provides both fixed and random effects estimates, giving a comprehensive evaluation of exposure-outcome effects by integrating individual Wald estimates.^[30]^ We conducted assessments using both IVW and MR-Egger methods, examining heterogeneity among instrumental variables with Cochran Q and Rucker Q statistics. A significant difference between these statistics (*P* ＜.05) indicates that the MR-Egger test is the preferred method for analyzing genetic associations between specific exposures and outcomes. We apply a random effects model if *P* ＜.05; otherwise, we use a fixed effects model.^[31]^ In MR-Egger regression, the intercept indicates the average pleiotropic effect of the instrumental variables. A significant deviation from zero suggests the presence of pleiotropy.^[32]^ Asymmetrical funnel plots may also indicate pleiotropy.^[33]^ The MR-PRESSO test is designed to identify and rectify outliers in IVW regression, encompassing the MR-PRESSO global, outlier, and distortion tests.^[34]^ Additionally, we conducted a “leave-one-out” sensitivity analysis to examine the influence of individual SNPs on the overall effect.^[35]^ All analyses utilized the TwoSampleMR and MR-PRESSO packages in R software (version 4.3.3).

## 3. Results

### 3.1. Identification of genetic instrumental variables

Identified 113 independent genetic variants associated with time spent watching television, 83 with leisure computer use and 7 with driving time. None of these variants showed significant correlations with COPD. Therefore, the chosen genetic variants are specifically correlated with COPD, with each variant’s *F*-statistic exceeding 10. During data harmonization, 3 palindromic SNPs was excluded with medium allele frequencies related to watching television, one to driving, and one to computer use (Tables S2–S4, Supplemental Digital Content, https://links.lww.com/MD/O710). Additionally, MR-PRESSO outlier detection led to the exclusion of 5 and 1 pleiotropic genetic variants for watching television and driving, respectively, listed in Table S5, Supplemental Digital Content, https://links.lww.com/MD/O710.

### 3.2. Univariate analysis

**Figure 2.**
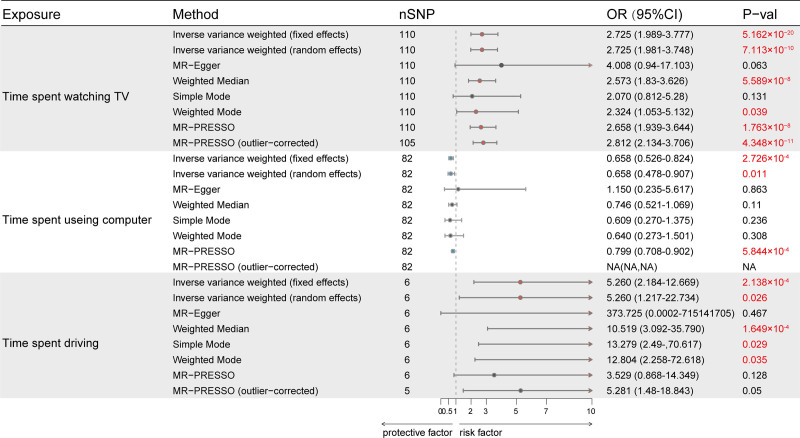
The causal association between leisure sedentary behaviors and COPD was estimated using different methods, including IVW (fixed effects and random effects), MR-Egger, Weighted Median, Simple Mode, Weighted Mode, MR-PRESSO, and MR-PRESSO (outlier-corrected). The X-axis displays odds ratios, with data displayed as OR and 95% CI. CI = confidence interval, COPD = chronic obstructive pulmonary disease, IVW = inverse-variance-weighted, OR = odds ratio.

Employing the IVW-fixed effects model, our analysis revealed that each SD increase (1.5 hours) in television watching time significantly increases the risk of COPD (OR = 2.725, 95% CI = 1.981–3.748, *P* = 5.162 × 10^−20^) (Fig. 2 and Table S6, Supplemental Digital Content, https://links.lww.com/MD/O710). Despite wide confidence intervals (CIs) in other mode analyses, the effect estimates remained similar, indicating a strong positive correlation between television watching time and COPD risk. In contrast, each SD increase (1.2 hours) in leisure computer use significantly decreases COPD risk (OR = 0.658, 95% CI = 0.526–0.824, *P* = 2.726 × 10^−4^). Results from the MR-PRESSO method also support this finding. Each SD increase in (1 hour) driving time significantly increases COPD risk (OR = 5.260, 95% CI = 2.184–12.669, *P = *2.138 × 10^−4^). However, due to the limited number of genetic variants for driving, the estimates for genetic correlations and CIs in sensitivity analyses were significant, providing insufficient evidence of a causal link.

### 3.3. Pleiotropy investigation

We employed Cochran *Q* and Rucker *Q* tests to evaluate the MR-IVW and MR-Egger analysis results, respectively (Table S7, Supplemental Digital Content, https://links.lww.com/MD/O710). These tests revealed heterogeneity in the genetic variants linked to time spent television watching, computer use, and driving. Due to this potential heterogeneity, employing the MR-IVW random effects model, we found significant associations between these activities and COPD risk: television watching (OR = 2.725, 95% CI = 1.989–3.777, *P* = 7.113 × 10^−10^), leisure computer use (OR = 0.658, 95% CI = 0.478–0.907, *P* = .011), and driving (OR = 5.260, 95% CI = 1.217–22.734, *P = *.026). No significant differences were observed between Cochran and Rucker Q values, suggesting balanced pleiotropy. Figures S1–S3, Supplemental Digital Content (https://links.lww.com/MD/O711), provide further visual checks for heterogeneity.

### 3.4. Multivariable analysis

**Figure 3.**
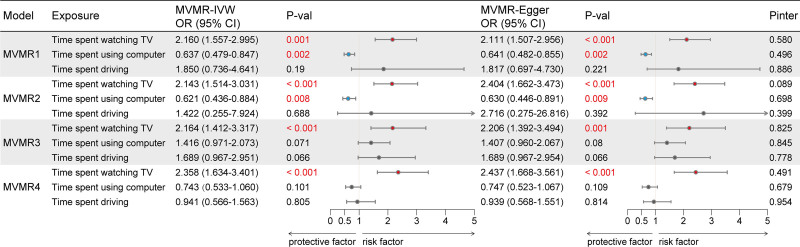
Summary univaribale MR estimates of leisure sedentary behaviors on COPD. MVMR 1, multivariable MR analysis adjusting for smoking initiation; MVMR 2, multivariable MR analysis adjusting for cigarettes smoked per day; MVMR 3, multivariable MRanalysis adjusting for years of schooling; MVMR 4, multivariable MR analysis adjusting for BMI. Pinter: *P*-value for intercept test of multivariable MR-Egger. On the X-axis, ORs are shown and data are represented as OR and 95% CI. BMI = body mass index, CI = confidence interval, COPD = chronic obstructive pulmonary disease, MR = Mendelian randomization, MVMR = multivariable mendelian randomization, OR = odds ratio.

In multivariable mendelian randomization (MVMR) analysis, after adjusting for potential confounders such as smoking initiation age, cigarettes smoked per day, years of schooling, and BMI, a significant positive causal association between television watching time and COPD risk persisted across various adjustments: adjusted for smoking initiation age (IVW: OR = 2.16, 95% CI = 1.557–2.995, *P* = .001); adjusted for cigarettes smoked per day (IVW: OR = 2.143, 95% CI = 1.514–3.031, *P* ＜.001); adjusted for years of schooling (IVW: OR = 2.164, 95% CI = 1.412–3.317, *P* ＜.001); and adjusted for BMI (IVW: OR = 2.358, 95% CI = 1.634–3.401, *P* ＜.001) (Fig. 3 and Table S8, Supplemental Digital Content, https://links.lww.com/MD/O710). Compared to MVMR IVW analysis, MVMR-Egger showed similar causal effect estimates, although with wider CIs. This consistency with univariate findings underscores the causal relationship’s robustness. Nonetheless, after adjusting for the aforementioned confounding variables, our MR results indicated that the previously observed negative correlation between leisure computer use and COPD risk became non in the univariate analyses of years of schooling and BMI, and even reversed to a positive correlation in the years of schooling analysis. Following adjustments, no significant association was found between driving time and COPD risk. Additionally, the MVMR-Egger intercept test (*P *> .05) provided limited evidence that the estimated effects of television watching, computer use, and driving time in the multivariable MR model were not substantially influenced by directional pleiotropy.

Consequently, we lack sufficient evidence to identify driving as a causative risk factor for COPD conclusively, nor can we definitively characterize leisure computer use as a protective factor against COPD, as this relationship could not be established in some sensitivity analyses, seemingly due to the influence of potential pleiotropic variants. In contrast, the positive association between leisure time spent watching television and increased COPD risk is consistently supported across various models, demonstrating robust evidence for this relationship.

## 4. Discussion

In this study, we employed MR approach to evaluate the potential causal effects of sedentary behaviors, including leisure time watching television, using computers, and driving on the risk of COPD. The results indicate a definitive causal link between a genetically predicted increase of 1.5 hours in leisure time television watching and an elevated risk of COPD. However, the evidence is insufficient to establish a genetic-based causal relationship between leisure time computer use or driving duration and the risk of developing COPD.

Cohort studies from Japan reveal that males engaging in over 4 hours of daily television watching face a 1.63-fold greater risk of mortality related to COPD than their counterparts who watch television for <2 hours.^[36]^ Similarly, European cohort studies demonstrate that individuals who sit for more than 6 hours per day are at a 1.33 times higher risk of developing COPD compared to those who sit for 2 hours or less.^[16]^ Physical inactivity, a consequence of sedentary behavior, can exacerbate systemic inflammation and impair lung function, both critical factors in COPD development.^[37–39]^

Extended sitting may negatively impact pulmonary ventilation and gas exchange efficiency and contribute to muscle mass degradation, further affecting respiratory efficiency.^[40–42]^ Furthermore, prolonged television watching is often associated with increased caloric intake and smoking, leading to obesity, another significant risk factor for COPD.^[14,15]^ For children aged 7 to 11 years, television watching and video game playing are associated with a 17% to 44% increased risk of overweight and a 10% to 61% increased risk of obesity.^[43]^ In adults, Brown et al^[44]^ found that among 8071 middle-aged women, those who reported sitting for more than 4.5 hours per day were significantly more likely to gain over 5 kg compared to those who sat for <3 hours daily. Consequently, public health measures and individual behavior changes are pivotal in COPD’s prevention and management.^[45]^

The principal advantage of this research lies in its capability to effectively mitigate the influence of confounding factors and reverse causality, a feat challenging to achieve in conventional observational studies. Utilizing a range of MR analytical approaches, including the IVW method and MR-Egger regression, we have substantially increased our results’ dependability and stability, thereby assuring our conclusions’ precision. Furthermore, our study predominantly relies on data from GWAS involving European populations, suggesting that the impact of ethnic diversity on our findings is likely limited, potentially enhancing their broader applicability.^[30]^

Nonetheless, there are certain constraints to our study. First, the exclusive use of GWAS data from European cohorts may restrict the extrapolation of our findings to diverse racial or geographical groups, thus potentially limiting the universality of our conclusions. For instance, participants in the UK Biobank had an average age of 57 years,^[20]^ a demographic known for spending significant time watching television. Therefore, these findings are not applicable to younger populations, whose viewing habits were not considered in the analysis. Second, given the wide array of COPD symptoms, more extensive subgroup analyses may be required in future research to more comprehensively discern the relationship between prolonged television watching and the various manifestations and severities of COPD. Third, the inherent assumption in MR analysis of lifelong exposure to specific risk factors could result in overestimating intervention impacts on COPD risks. Also, our analyses exclusively targeted leisure sedentary exposures, thus omitting non-leisure sedentary behaviors. Given the extensive sample size in the MR analysis, we are confident that the estimated effects closely approximate the actual values.

## 5. Conclusion

In conclusion, the MR analysis from this study reveals that a genetically predicted increase of 1.5 hours in television watching duration is significantly associated with a higher risk of COPD. These findings corroborate the results of conventional observational epidemiological studies and suggest that public health policies and interventions aimed at reducing sedentary behaviors, such as excessive television viewing, may contribute to the prevention of COPD development and progression.

## Acknowledgments

The authors express their gratitude to BioRender.com for providing materials to create the experimental schedule. Additionally, the authors would like to thank the researchers who contributed to the Genome-Wide Association Study (GWAS) data.

## Author contributions

**Conceptualization:** Ying Li.

**Data curation:** Lianying Guo, Mengqi Jiang, Yuqing Cheng.

**Software:** Ying Li, Lianying Guo, Xu Feng.

**Supervision:** Ye Yu, Lili Deng, Zhuo Zhang.

**Validation:** Yuqing Cheng, Qingyi Zhou.

**Writing – original draft:** Ying Li.

**Writing – review & editing:** Ye Yu, Lu Sun, Zhuo Zhang.

## Supplementary Material


