# Supplementary material for: Prolonged leisure time television watching as a risk factor for chronic obstructive pulmonary disease: Insights from Mendelian randomization
Source: Medicine (Baltimore). 2025 Apr 18;104(16):e42142. doi: 10.1097/MD.0000000000042142 (PMC12014096; doi:10.1097/MD.0000000000042142)
Supplement: Supplementary file 2 [file medi-104-e42142-s002.pdf]

Figrue S1 Leisure time watching television and COPD in scatter plot, individual removal sensitivity analysis plot, forest plot and funnel plot

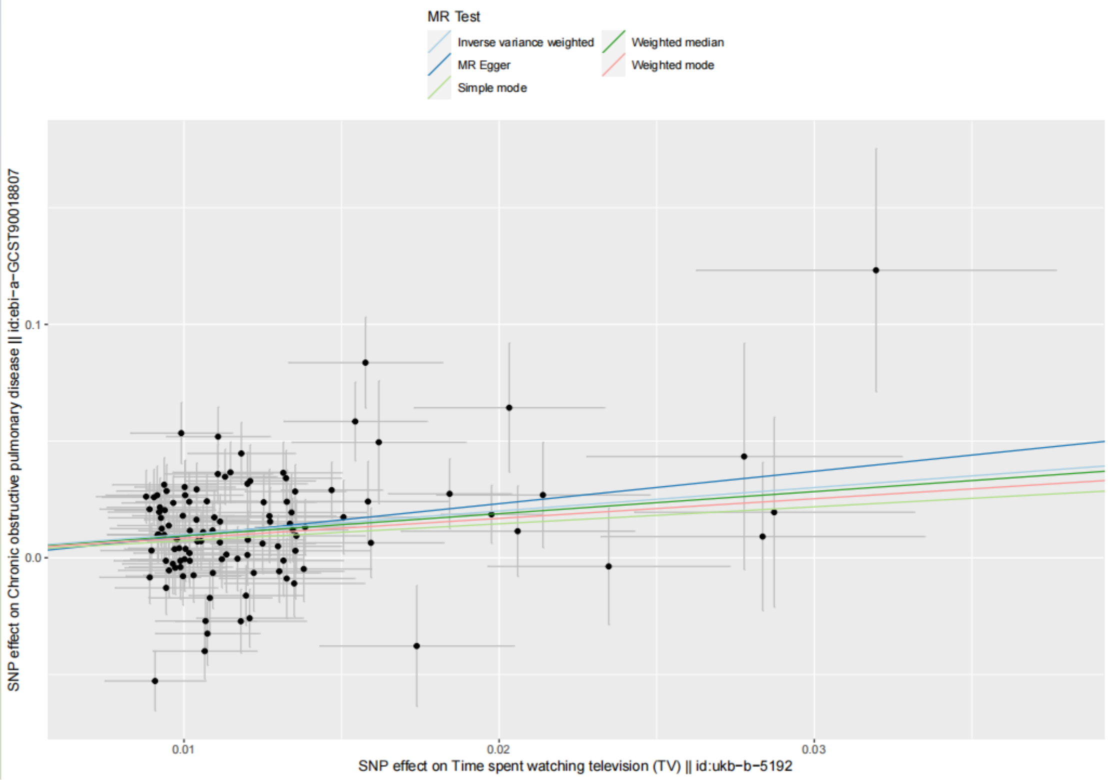

scatter plot

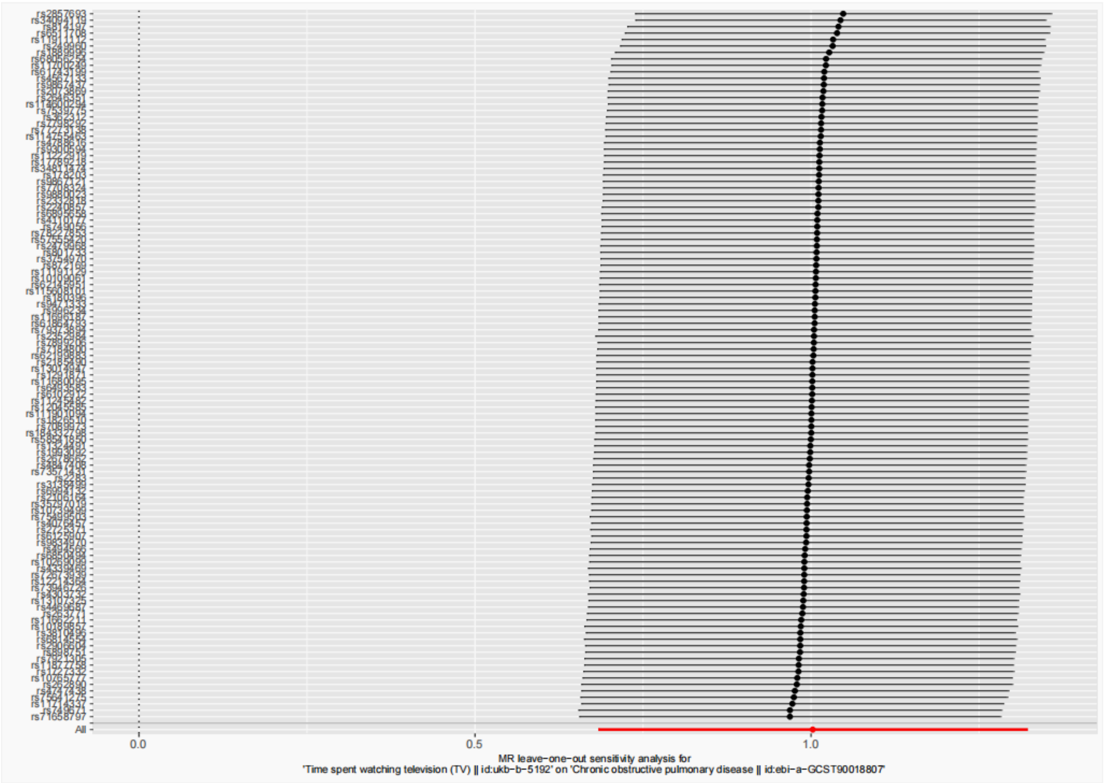

individual removal sensitivity analysis plot

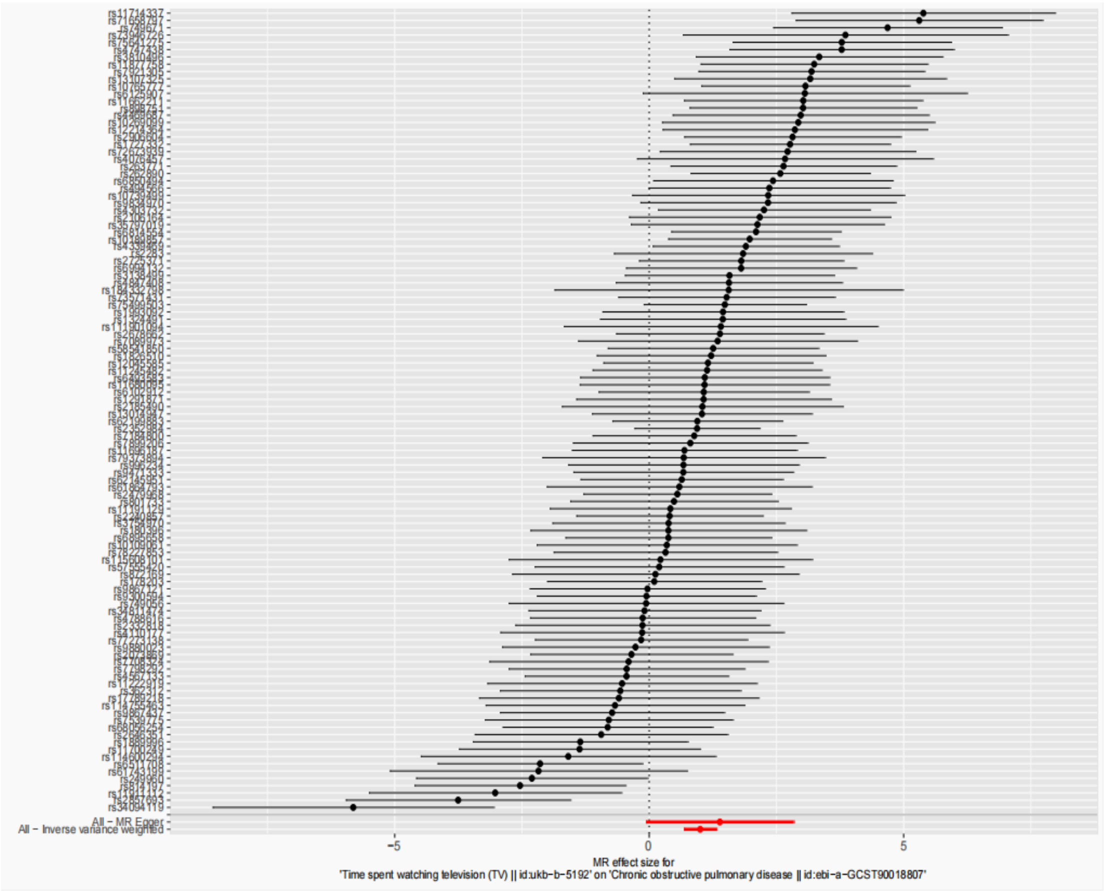

forest plot

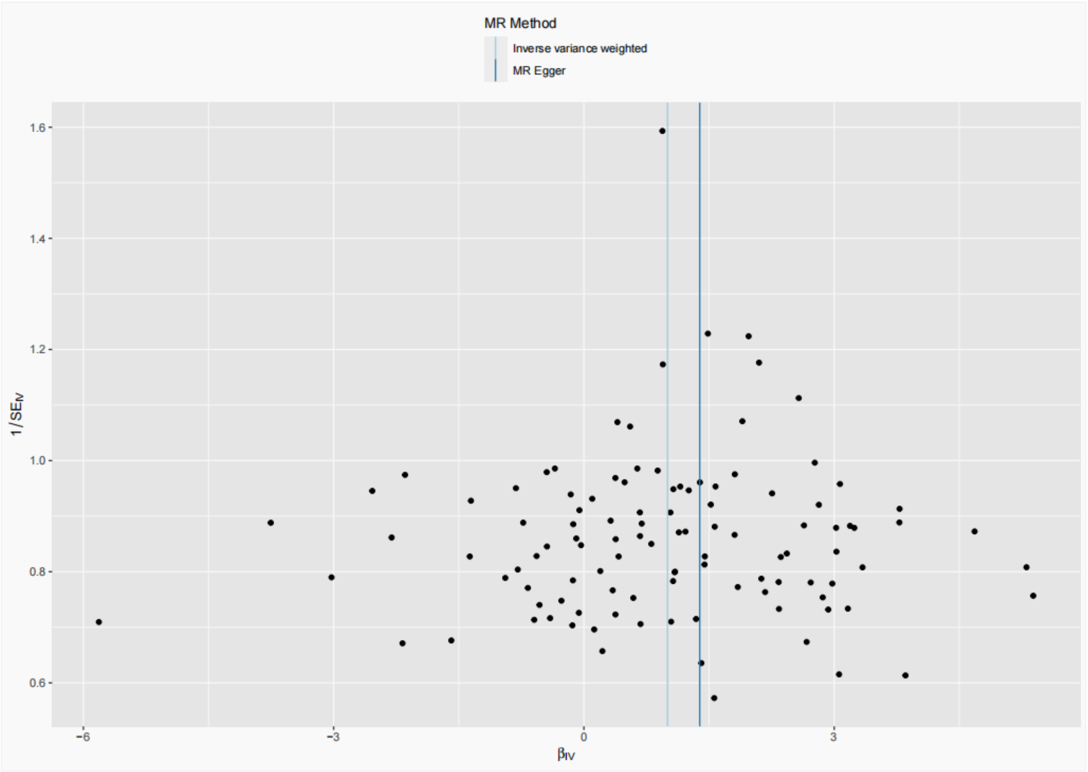

funnel plot

Figrue S2 Leisure time using computer and COPD in scatter plot, individual removal sensitivity analysis plot, forest plot and funnel plot

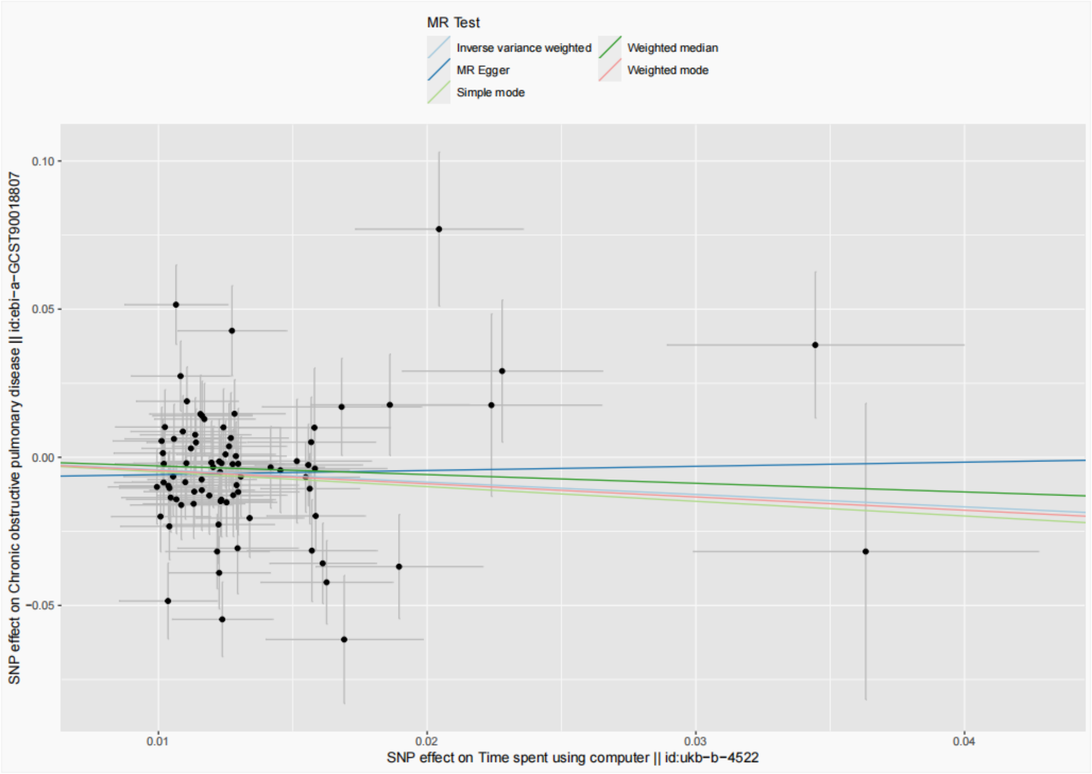

scatter plot

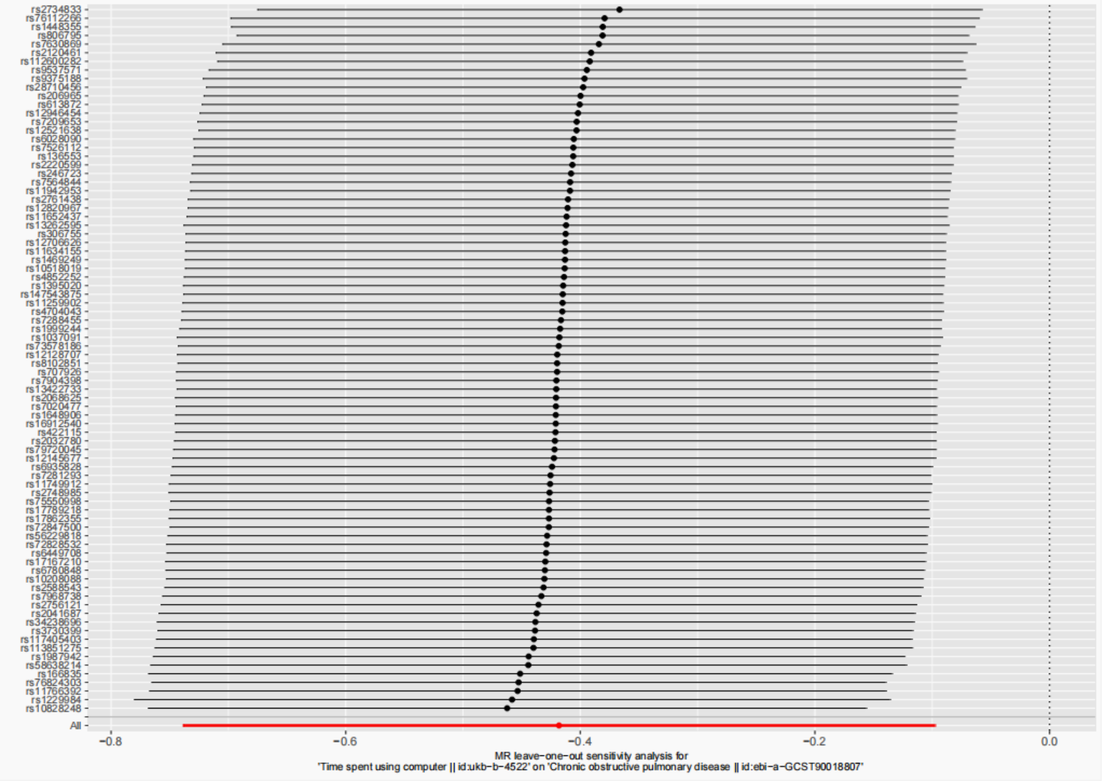

individual removal sensitivity analysis plot

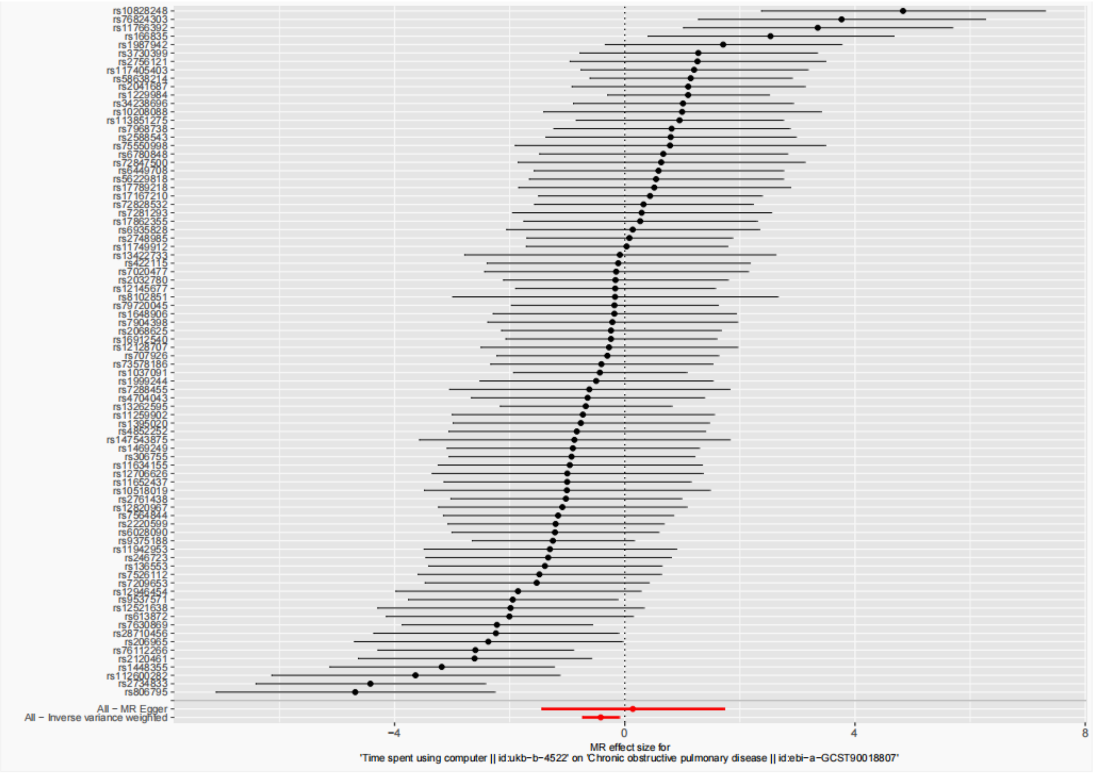

forest plot

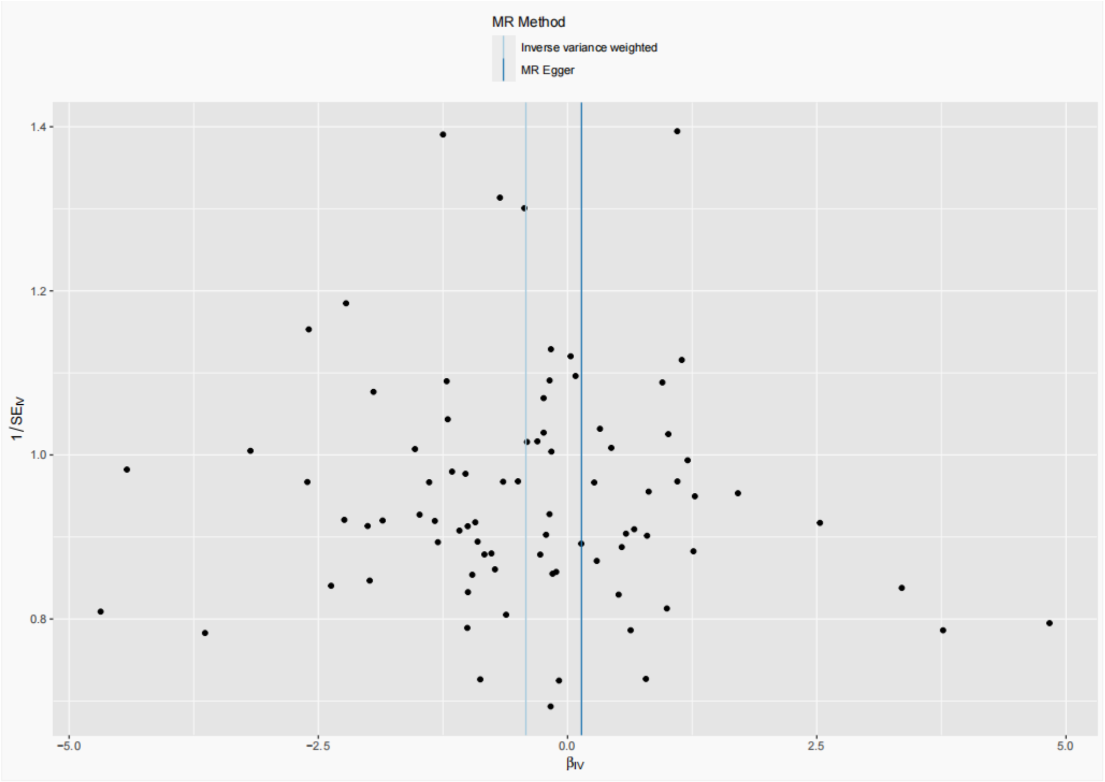

funnel plot

Figrue S3 Leisure time driving and COPD in scatter plot, individual removal sensitivity analysis plot， forest plot and funnel plot

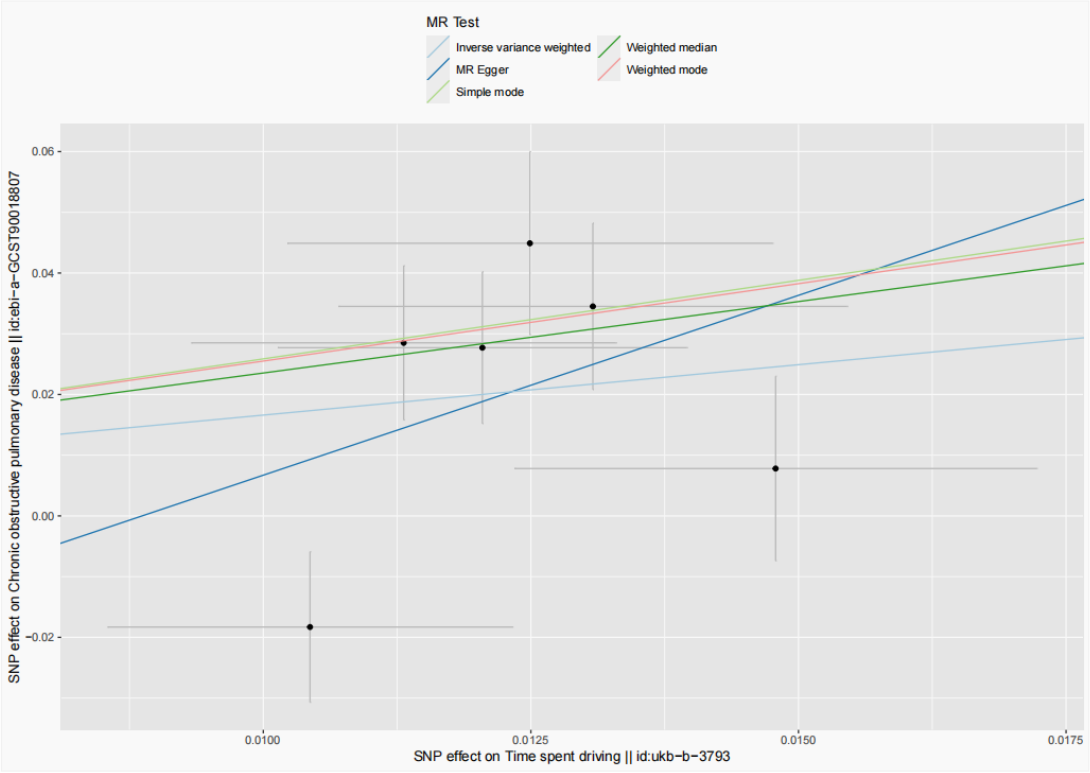

scatter plot

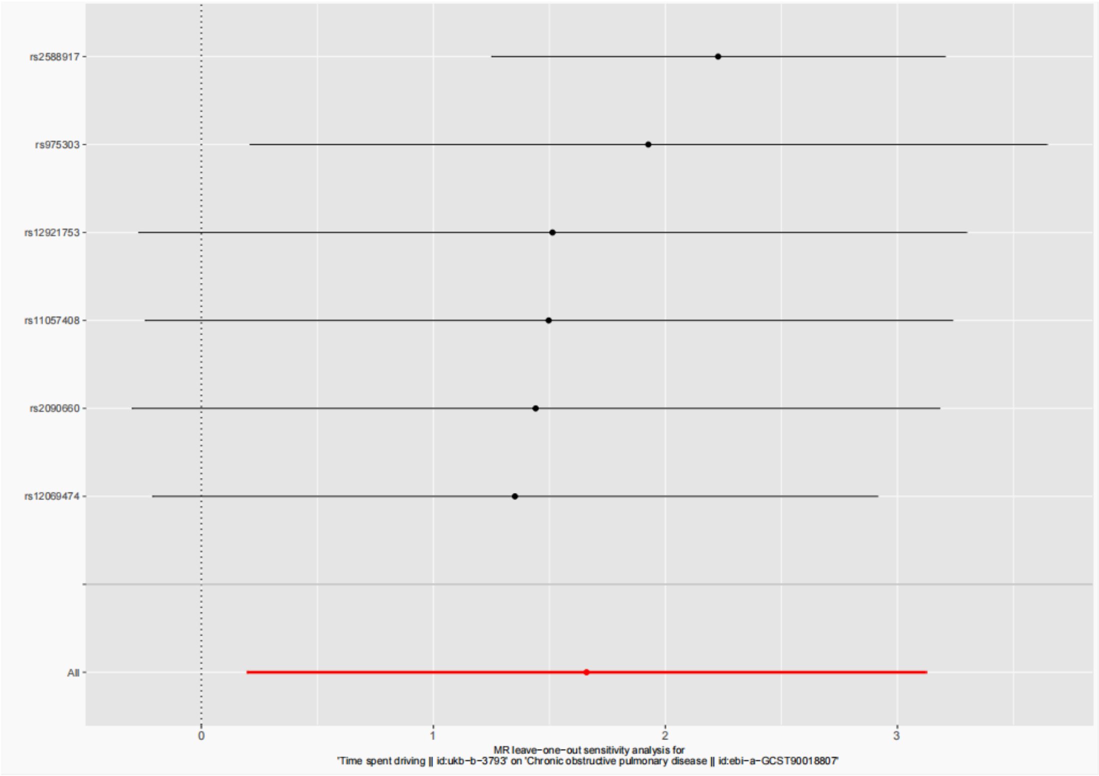

individual removal sensitivity analysis plot

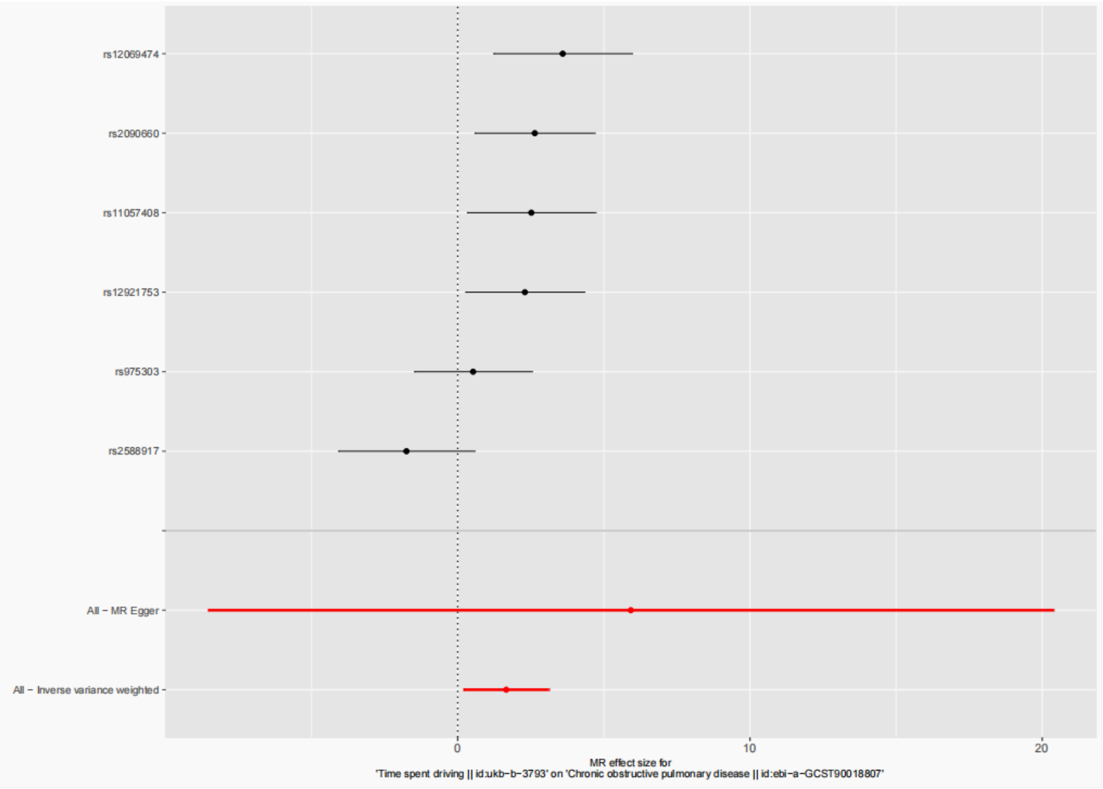

forest plot

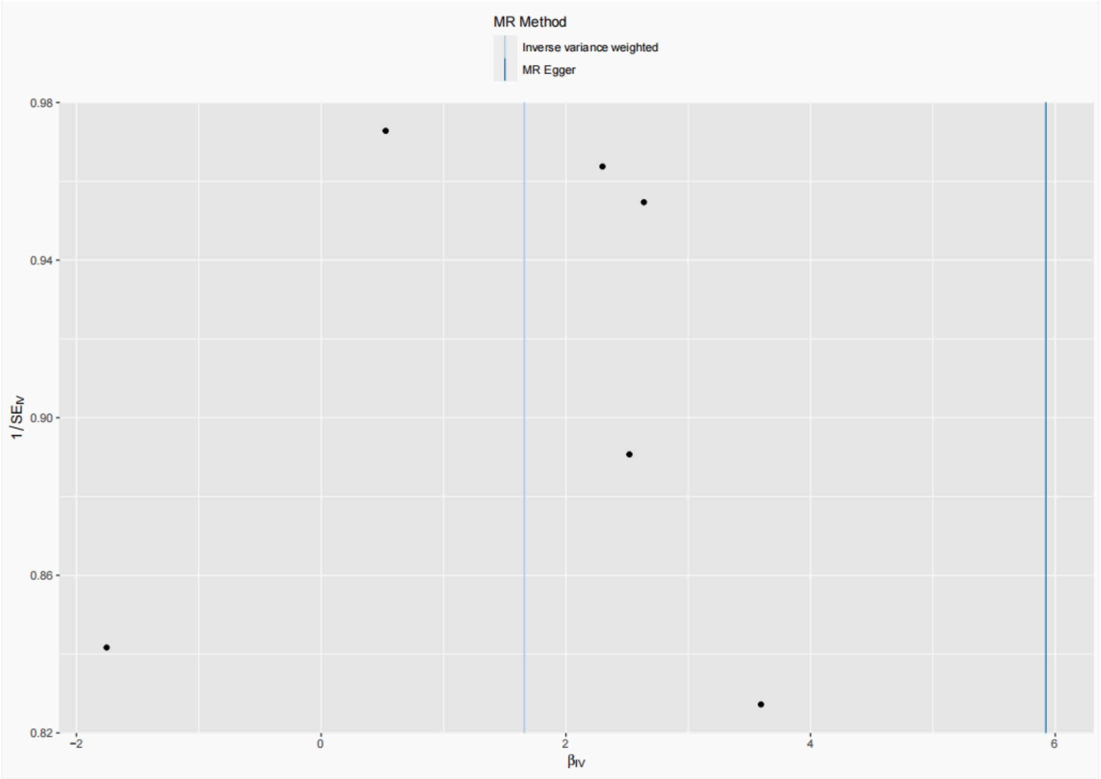

funnel plot
